# Association of corneal nerve parameters with nerve abnormalities and neuropathic pain in prediabetes and type 2 diabetes: the Maastricht Study

**DOI:** 10.1007/s00125-026-06676-8

**Published:** 2026-02-10

**Authors:** Mette K. Borbjerg, Sara Mokhtar, Nadia Sutedja, Annemarie Koster, Carsten D. Mørch, Tos T. J. M. Berendschot, Nicolaas Schaper, Niels Ejskjaer, Johan Røikjer

**Affiliations:** 1 Steno Diabetes Center North Denmark, Aalborg University Hospital, Gistrup, Denmark; 2 Translational Pain Neuroscience and Precision Health, Department of Health Science and Technology, Aalborg University, Aalborg, Denmark; 3 Department of Internal Medicine, CARIM School for Cardiovascular Diseases, Maastricht University, Maastricht, the Netherlands; 4 University Eye Clinic Maastricht, School of Mental Health and Neuroscience, Maastricht University, Maastricht, the Netherlands; 5 Department of Clinical Neurophysiology, Maastricht University Medical Centre+, Maastricht, the Netherlands; 6 Department of Social Medicine, CAPHRI Care and Public Health Research Institute, Maastricht University, Maastricht, the Netherlands; 7 Center for Neuroplasticity and Pain (CNAP), Department of Health Science and Technology, Aalborg University, Aalborg, Denmark; 8 Department of Internal Medicine, CARIM School for Cardiovascular Diseases, Maastricht University Medical Centre+, Maastricht, the Netherlands; 9 Department of Endocrinology, Aalborg University Hospital, Gistrup, Denmark; 10 Department of Clinical Medicine, Aalborg University Hospital, Aalborg, Denmark

**Keywords:** Cornea, Corneal confocal microscopy, Diabetic polyneuropathy, Neuropathy, Pain

## Abstract

**Aims/hypothesis:**

Corneal confocal microscopy is a valuable technique for assessing neuropathy; however, whether it can distinguish painful from painless neuropathy remains uncertain and existing evidence is based on the results of smaller studies. This study assessed the association of corneal nerve parameters with abnormalities identified by electromyography (EMG) and neuropathic pain in a large population with and without (pre)diabetes.

**Methods:**

In this study we included cross-sectional data for 3425 participants from the Maastricht Study. Wide-field corneal confocal microscopy (WF-CCM) was performed using fully automated analysis of three corneal nerve parameters: corneal nerve branch density (CNBD), corneal nerve fibre density (CNFD) and corneal nerve fibre length (CNFL). An axonal degeneration composite score comprising compound muscle action potential amplitudes (peroneal and tibial) and the sensory nerve action potential amplitude of the sural nerve was created by categorising EMG amplitudes as normal or indicating minor (≤10th percentile), moderate (≤5th percentile) or severe (≤2.5th percentile) abnormalities. Neuropathic pain was determined as a modified Douleur Neuropathique en 4 Questions (DN4) interview score ≥3.

**Results:**

The mean age of the participants was 59.2 years; 51.6% were female, 15% had prediabetes (defined as impaired fasting glucose, impaired glucose tolerance or both) and 19% had type 2 diabetes. The median diabetes duration was 3.0 years. Regression analyses revealed statistically significant associations between the axonal degeneration EMG score and WF-CCM parameters (CNFL: β=−0.51 [95% CI −0.78, −0.24]; CNFD: β=−1.56 [95% CI −3.04, −0.08]; CNBD: β=−3.08 [95% CI −5.51, −0.64]; all *p*<0.05) but no statistically significant associations between neuropathic pain and WF-CCM parameters (CNFL: β=−0.06; CNFD: β=−1.15; CNBD: β=−0.22; all *p*>0.1).

**Conclusions/interpretation:**

The study found associations between the axonal degeneration EMG score and WF-CCM, but no associations were observed between neuropathic pain and WF-CCM parameters, suggesting that WF-CCM has limited value in assessing neuropathic pain.

**Graphical Abstract:**

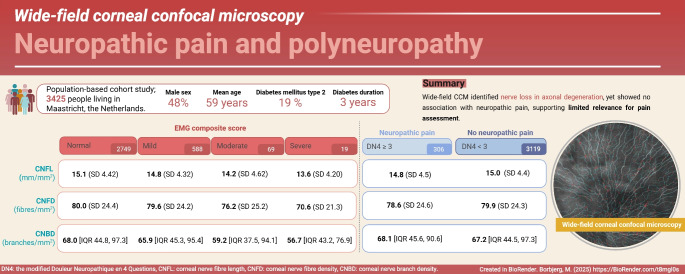

**Supplementary Information:**

The online version contains peer-reviewed but unedited supplementary material available at 10.1007/s00125-026-06676-8.


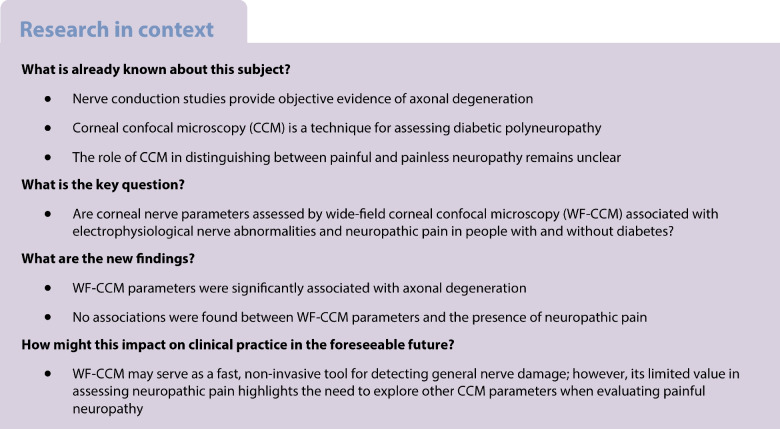


## Introduction

Polyneuropathy affects up to 50% of people with diabetes, leading to increased morbidity and reduced quality of life [1–6]. To reduce the increased morbidity, early diagnosis is necessary to enable early therapeutic interventions [7]. Here screening is of utmost importance, as the symptoms of sensory loss are insidious and often stem from progressive axonal degeneration, which is a key feature of large-fibre axonal polyneuropathy [1, 8]. Painful polyneuropathy is seen in around 10–30% of individuals with diabetic polyneuropathy (DPN) and is thought to be a sign of small-fibre neuropathy [9]. The condition is associated with reduced quality of life and higher levels of anxiety symptoms compared with painless DPN [4, 7]. The underlying mechanisms of neuropathic pain in DPN are not fully understood, but mechanisms such as reduced inhibition of spinal neurones from descending pathways and altered peripheral blood flow may play a role, while the neuronal hyperexcitability may be related to alterations in ion channel and receptor expression, reactive metabolites, neurotransmitter release and inflammatory factors [10, 11].

Recent advances in the diagnosis of DPN have enabled assessment of small nerve fibre structure and function [12]. In vivo corneal confocal microscopy (CCM) allows visualisation and evaluation of the sub-basal plexus [13]. When performing CCM, corneal nerve fibre length (CNFL), corneal nerve fibre density (CNFD) and corneal nerve branch density (CNBD) are often used to describe the corneal nerve conditions. CNFL has been found to be lower in individuals with both prediabetes and diabetes [14–17] and to be associated with DPN [17–19].

The ability of CCM to distinguish between painful and painless DPN has been investigated, but mainly in smaller studies, with contradictory findings reported in the current literature. The studies reported either no difference or differences in specific CCM parameters between painful and painless DPN [20–22]. Püttgen et al found enhanced CNBD in people with painful DPN compared with those with painless DPN, consistent with findings from epidermal nerve fibres [21, 23]. By visualisation of the sub-basal plexus, knowledge regarding the underlying mechanisms of neuropathic pain in DPN may be acquired.

For this reason, our aim was to assess whether corneal nerve parameters (CNFL, CNFD, CNBD, fractal dimension and tortuosity) are associated with (1) the degree of abnormalities identified by electromyography (EMG) and (2) the presence of neuropathic pain.

## Methods

### Study population and design

We used data from the Maastricht Study, an observational prospective population-based cohort study. The rationale and methodology have been described previously [24]. In brief, the study focuses on the aetiology, pathophysiology, complications and comorbidities associated with type 2 diabetes mellitus and is characterised by an extensive phenotyping approach. All individuals aged between 40 and 75 years living in the southern part of the Netherlands were eligible for participation. Participants were recruited through mass media campaigns and from municipal registries and the regional Diabetes Patient Registry via mailings. Recruitment was stratified according to known type 2 diabetes status, with oversampling of individuals with type 2 diabetes to increase the statistical power to identify any differences between individuals with and without type 2 diabetes [24]. The representativeness of the cohort relative to the underlying source population was monitored continuously throughout recruitment, as previously described by Schram et al, ensuring alignment with regional postal code distributions [24].

**Fig. 1:**
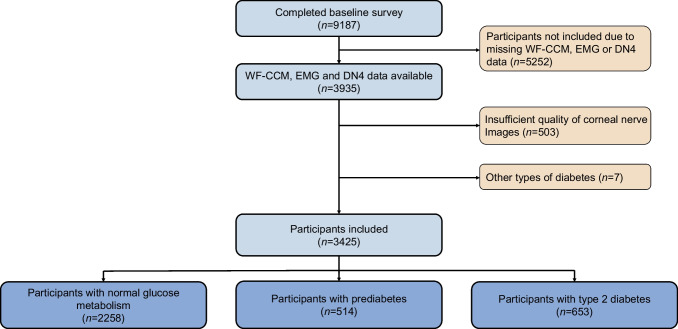
Overview of selection of the study population

The present study includes cross-sectional data for 9187 participants who completed the baseline survey between November 2010 and October 2020. The examinations for each participant were performed within a time window of 3 months. Wide-field corneal confocal microscopy (WF-CCM) measurements were performed between April 2013 and October 2020 (*n*=4317). Individuals who participated in the Maastricht Study before April 2013 (*n*=974) were re-invited for a ‘catch-up visit’, as described previously [14]. For the current analyses, participants were included if WF-CCM, EMG and modified Douleur Neuropathique en 4 Questions (DN4) interview data were available (*n*=3935). Participants with insufficient quality of WF-CCM images (*n*=503) or with a diabetes type other than type 2 diabetes (*n*=7) were subsequently excluded, resulting in a final sample of 3425 individuals (Fig. 1).

The study was approved by the institutional medical ethical committee of Maastricht University Medical Center (MUMC+) and Maastricht University (NL31329.068.10) and the Minister of Health, Welfare and Sports of the Netherlands (permit 131088-105234-PG). All participants gave written informed consent.

### Assessment of CCM measurements

We performed WF-CCM using a Heidelberg retina tomograph III (HRT3) with Rostock cornea module (Heidelberg Engineering, Heidelberg, Germany) to image the corneal nerves [25] of the left eye only for logistical reasons. The methodology for WF-CCM imaging has been described previously [14]. Both eyes were anaesthetised using oxybuprocaine hydrochloride (0.4 mg/ml) and lubricated with eye gel (carbomer 2 mg/g) to ensure optimal image acquisition. Trained research assistants imaged the sub-basal nerve plexus layer in the central part of the cornea. We assessed multiple recordings for areas of 400×400 μm (384×384 pixels, 8-bit) assembled using a composite algorithm as previously described [25]. Real-time mapping was performed on an area of up to 1600×1600 µm (1536×1536 pixels, 8-bit) that partially included the inferior whorl in some of the composite images. We used the U-net-based convolutional neural network to fully automatically trace the corneal nerves [26] and analysed the following indices: CNBD (number of bifurcation points [branching points] per mm^2^), CNFD (total number of corneal nerve fibres [including both main fibres and branches] per mm^2^), CNFL (total length of corneal nerve fibres in mm [both main fibres and branches] per mm^2^), corneal nerve fractal dimension (quantification of the nerve structure complexity) and corneal nerve tortuosity (a dimensionless measure of the curvature of nerve fibres, calculated as the ratio of the actual nerve path length to the straight-line distance between its endpoints). Composite segmented images were reviewed manually and images were considered to be usable (*n*=3758) if 50% or more of the total captured area was of good quality [14].

### Assessment of nerve function

The method for assessment of lower-leg nerve function has been described in detail previously [27]. Skin temperature was measured on the dorsal surface of the foot. In the nerve conduction studies, we measured the compound muscle action potential (CMAP) of the extensor digitorum brevis muscle (peroneal nerve) and the abductor hallucis muscle (tibial nerve), together with the sensory nerve action potential (SNAP) of the sural nerve. Amplitudes were recorded from baseline to negative peak and from onset to peak for compound muscle and sensory action potential, respectively. All data were recorded using a Medelec Synergy electromyography apparatus (version 15.0, Viasys Healthcare) [27].

All EMG data were reviewed and values above or below 3 SD from the mean were manually reviewed by an expert. The method for data processing of EMG data has been described in detail previously [27].

Nerve function was assessed by comparing participants’ measures with normative data stratified by sex and age group (40–50 years, 50–60 years, 60–70 years, and 70+ years). The normative data were based on data from the Maastricht Study (*N*=9187), excluding individuals without EMG (*n*=709), those with absent tibial, peroneal or sural nerve amplitude (*n*=929), those missing skin temperature data (*n*=382) or with a skin temperature <32°C (*n*=165) and individuals with diabetes or prediabetes (*n*=3440).

To encompass all measures of nerve function, we calculated an EMG composite score [28] including the following measures: CMAP amplitude, nerve conduction velocity and distal motor latency of the peroneal and tibial nerves, and SNAP amplitude and nerve conduction velocity of the sural nerve. Each measure was first categorised as 0 (normal), 1 (mild abnormality, ≤10th percentile), 2 (moderate abnormality, ≤5th percentile) or 3 (severe abnormality, ≤2.5th percentile). The EMG composite score was then derived by summing these categorical scores and dividing by the number of measures.

Applying the same methods, a composite score focusing on axonal degeneration was calculated based on the following measures: CMAP amplitude of the peroneal and tibial nerves and SNAP amplitude of the sural nerve.

### Assessment of neuropathic pain

The presence of neuropathic pain symptoms was determined using the Dutch version of the DN4 interview [29]. Individuals were grouped into neuropathic pain (DN4 interview score ≥3) or no neuropathic pain (DN4 interview score <3). Participants without DN4 interview answers (*n*=42) were excluded. Data on medication prescription were collected for medication related to neuropathic pain (gabapentin, pregabalin, duloxetine, amitriptyline, nortriptyline and carbamazepine).

### Assessment of glucose metabolism status

After an overnight fast, participants underwent a standardised seven-point OGTT: venous samples were collected at baseline and 15, 30, 45, 60, 90 and 120 min after ingestion of a 75 g glucose drink. All participants underwent an OGTT except those who used insulin or had a fasting plasma glucose concentration above 11.0 mmol/l. Based on medication use, fasting plasma glucose and 2 h post-load glucose, glucose metabolism status was determined as normal glucose metabolism, prediabetes (defined as impaired fasting glucose, impaired glucose tolerance or both), type 2 diabetes or other types of diabetes (including type 1 diabetes) according to the WHO 2006 criteria [30]. Individuals with other types of diabetes were not included in this study.

Fasting plasma glucose levels (mmol/l) and HbA_1c_ levels (mmol/mol and %) were determined in venous plasma samples collected after an overnight fast. The duration of diabetes in individuals with type 2 diabetes was assessed using a questionnaire [24].

### Covariates

As described previously [24], basic demographic and anthropometric characteristics were measured using standardised protocols. Information on smoking status (never, former, current), alcohol use (none, low, high), history of cancer, history of ocular disorders (corneal diseases or uveitis) and use of contact lenses was collected by questionnaire. Biological sex was self-reported by participants at enrolment. During a physical examination weight (kg), height (cm) and waist circumference (cm) were assessed in intervals of 0.5 kg or 0.1 cm, BMI (kg/m^2^) was calculated based on body weight and height, and office BP (mmHg) was measured. A fasting venous blood sample was collected to determine the lipid profile and serum creatinine level.

### Data analysis

Population characteristics are reported for the total study population and by neuropathic pain status using the appropriate descriptive statistics. All analyses were performed using R statistical software (version 4.1.2; R Foundation for Statistical Computing, Vienna, Austria).

Normality was assessed using a QQ plot and density plot. Non-normal distribution was observed within the subgroups defined by EMG abnormality and neuropathic pain status, and statistical differences was assessed using a Mann–Whitney *U* test. Comparisons were performed within each level of nerve conduction abnormality (no, mild, moderate or severe), comparing participants with and without neuropathic pain.

To examine the association between determinants (EMG abnormality or neuropathic pain) and corneal nerve parameters, linear regression analysis was performed on the total population, and additional analyses were performed stratified by glucose metabolism status, as described in the electronic supplementary material (ESM) Methods. All associations are expressed as unstandardised regression coefficients (β) with corresponding 95% CI in Tables 2, 3 and 4. Data precision is expressed as the CI width.

#### Nerve function

Associations between the degree of abnormalities identified by EMG (none, mild, moderate or severe) and corneal nerve parameters (CNFD, CNFL, CNBD, corneal nerve fractal dimension and tortuosity) were assessed using multivariable linear regression. The crude model examined EMG abnormality and corneal nerve parameters. Model 1 was adjusted for CCM lag time. Model 2 was further adjusted for age, sex, BMI, height and skin temperature. In a secondary analysis, the EMG composite score was substituted by the axonal degeneration composite score.

#### Neuropathic pain

Associations between neuropathic pain (yes/no) and corneal nerve parameters were assessed using multivariable linear regression. The crude model examined neuropathic pain and corneal nerve parameters. Model 1 was adjusted for CCM lag time, model 2 was additionally adjusted for age, sex, BMI and height, and model 3 was further adjusted for degree of EMG abnormalities and skin temperature.

Unless otherwise stated, interpretation is based on the model 2 specified for each analysis.

#### Additional analyses

To ensure the robustness of our findings, we performed several additional analyses, as described in ESM Methods. These included sensitivity analyses that excluded participants with factors that could potentially influence the outcome (i.e. high alcohol intake, history of cancer and severely reduced kidney function [eGFR <30 ml/min per 1.73 m^2^]), participants with a catch-up visit and those with newly diagnosed type 2 diabetes. Additional sensitivity analyses included associations between corneal nerve fibre parameters and markers of axonal degeneration (composite amplitude score), sural nerve amplitude (continuous) and tibial nerve amplitude (continuous). Furthermore, we evaluated the impact of pain medications on associations between neuropathic pain and corneal nerve parameters. Finally, to address the non-normally distributed residuals observed in the primary linear regression models, we performed a sensitivity analysis that excluded influential observations, defined as individual observations with absolute standardised residuals >2. Because no heteroskedasticity was detected, linear regression remained appropriate for the main analyses; the final sensitivity analysis was conducted solely to assess robustness.

## Results

A total of 3425 individuals were included in the study. Figure 1 provides an overview of the selection of the study population; baseline characteristics of included vs excluded individuals are presented in ESM Table 1. Demographic characteristics are shown in Table 1; 51.6% of participants were female and mean age was 59.2 years. There were 514 individuals with prediabetes and 653 with type 2 diabetes mellitus; the median type 2 diabetes duration was 3.0 years*.* Demographic characteristics stratified by glucose metabolism status are described in ESM Tables 2–4.

**Table 1:** General study population characteristics

Variable	Total (*N*=3425)	Neuropathic pain (*N*=306)	No neuropathic pain (*N*=3119)
Age (years)	59.2 ± 8.7	61.0 ± 8.2	59.0 ± 8.8
Female sex^a^	1769 (51.6)	169 (55.2)	1600 (51.3)
Race and ethnicity: White^a^	3383 (98.8)	301 (98.4)	3082 (98.8)
Fasting plasma glucose (mmol/l)	5.3 (4.9, 6.0)	5.7 (5.1, 6.9)	5.3 (4.9, 5.9)
HbA_1c_ (mmol/mol)	36.0 (33.0, 41.0)	39.0 (35.0, 45.0)	36.0 (33.0, 40.0)
HbA_1c_ (%)	5.4 (5.2, 5.9)	5.7 (5.4, 6.3)	5.4 (5.2, 5.8)
Glucose metabolism			
Normal	2258 (65.9)	145 (47.4)	2113 (67.7)
Prediabetes	514 (15.0)	48 (15.7)	466 (14.9)
Type 2 diabetes	653 (19.1)	113 (36.9)	540 (17.3)
Diabetes duration (years)	3.0 (0, 8.0)	2.0 (0, 8.8)	3.0 (0, 8.0)
Taking diabetes medication	445 (13.0)	86 (28.1)	359 (11.5)
BMI (kg/m^2^)	26.7 ± 4.3	28.0 ± 4.9	26.5 ± 4.2
Waist circumference(cm)	94.1 ± 13.1	98.3 ± 14.9	93.6 ± 12.9
Office systolic BP (mmHg)	133 ± 17.6	134 ± 17.5	132 ± 17.6
Office diastolic BP (mmHg)	75.4 ± 9.9	75.7 ± 10.2	75.4 ± 9.9
Taking antihypertensive medication	1160 (33.9)	163 (53.3)	997 (32.0)
Serum HDL-cholesterol (mmol/l)	1.6 ± 0.5	1.5 ± 0.5	1.6 ± 0.5
Serum LDL-cholesterol (mmol/l)	3.05 ± 1.0	2.90 ± 1.0	3.1 ± 1.0
Serum total cholesterol (mmol/l)	5.2 ± 1.1	5.1 ± 1.1	5.2 ± 1.1
eGFR (ml/min per 1.73 m^2^)^b^	88.8 (79.2, 97.9)	85.8 (77.2, 95.1)	89.1 (79.2, 98.1)
Smoking category			
Current	427 (12.5)	63 (20.6)	364 (11.7)
Former	1663 (48.6)	159 (52.0)	1504 (48.2)
Never	1335 (39.0)	84 (27.5)	1251 (40.1)
Alcohol consumption status^c^			
High	737 (21.5)	62 (20.3)	675 (21.6)
Low	2068 (60.4)	167 (54.6)	1901 (60.9)
None	619 (18.1)	77 (25.2)	542 (17.4)
Missing	1 (0)	0 (0)	1 (0)

Data are *n* (%), mean ± SD or median (IQR) as appropriate based on variable type and distribution

Neuropathic pain was defined as a DN4 interview score ≥3

^a^Sex and race/ethnicity were self-reported by participants at enrolment

^b^eGFR was calculated using the CKD-EPI equations (with both serum creatinine and cystatin C)

^c^High alcohol consumption was defined as >7 units/week for women and >14 units/week for men

**Fig. 2:**
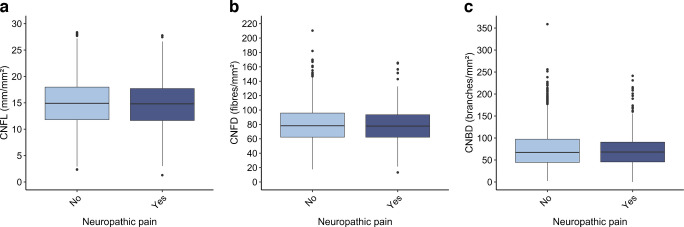
Results from the total population for (**a**) CNFL, (**b**) CNFD and (**c**) CNBD grouped by neuropathic pain status. Boxplots show median (horizontal line), IQR (box), and whiskers extending to 1.5×IQR; dots represent outliers. Pairwise comparisons between the groups with and without pain were performed using the Mann–Whitney *U* test: no statistically significant differences were observed

In the total population, the prevalence of neuropathic pain was 8.9%. In the group with neuropathic pain, 31.1% had EMG abnormalities, with 1.6% having severe abnormalities; in those without neuropathic pain, these percentages were 18.6% and 0.5%, respectively (ESM Table 5). In terms of corneal nerve data, the CNFL was 14.8±4.5 and 15.0±4.4 mm/mm^2^, the CNFD was 78.6±24.6 and 79.9±24.3 fibres/mm^2^, and the CNBD was 68.1 (IQR 45.6, 90.6) and 67.2 (IQR 44.5, 97.3) branches/mm^2^, respectively, in participants with and without neuropathic pain. Median values and IQRs are shown in Fig. 2a–c. Corneal nerve data grouped by neuropathic pain and the degree of abnormalities identified by EMG are shown in ESM Tables 5 and 6.

**Fig. 3:**
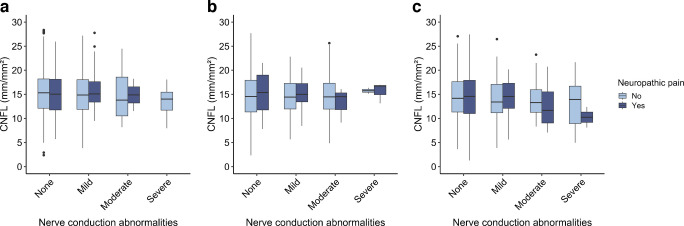
CNFL across groups stratified by degree of EMG abnormalities and pain status for participants with normal glucose metabolism (**a**), prediabetes (**b**) and type 2 diabetes mellitus (**c**). Nerve conduction abnormalities on EMG were classified into four severity levels: none, mild, moderate and severe. No participants with normal glucose metabolism had severe EMG abnormalities in combination with neuropathic pain. Boxplots show median (horizontal line), IQR (box), and whiskers extending to 1.5×IQR; dots represent outliers. Pairwise comparisons between the groups with and without pain were performed using the Mann–Whitney *U* test: no statistically significant differences were observed

**Fig. 4:**
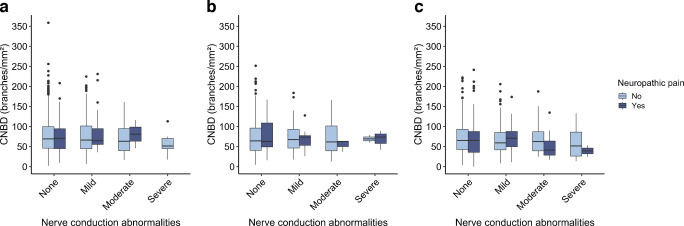
CNBD across groups stratified by degree of EMG abnormalities and pain status for participants with normal glucose metabolism (**a**), prediabetes (**b**) and type 2 diabetes mellitus (**c**). Nerve conduction abnormalities on EMG were classified into four severity levels: none, mild, moderate and severe. No participants with normal glucose metabolism had severe EMG abnormalities in combination with neuropathic pain. Boxplots show median (horizontal line), IQR (box), and whiskers extending to 1.5×IQR; dots represent outliers. Pairwise comparisons between the groups with and without pain were performed using Mann–Whitney *U* test: no statistically significant differences were observed

Corneal nerve data in relation to neuropathic pain, stratified by degree of abnormality identified by EMG and glucose metabolism status, are shown in Fig. 3a–c and Fig. 4a–c. As shown in the boxplots, the median CNFL for the normal glucose metabolism and prediabetes groups was similar across different degrees of EMG abnormalities and similar between the groups with and without neuropathic pain. In type 2 diabetes, a lower median CNFL was seen for the neuropathic pain group than in the group without neuropathic pain, particularly in participants with severe EMG abnormalities and to a lesser extent in those with moderate EMG abnormalities, but these differences were not statistically significant (all *p*>0.20). The median CNBD was similar across the different degrees of EMG abnormalities in the group without neuropathic pain. For the neuropathic pain group, the median CNBD was generally lower in individuals with type 2 diabetes and moderate and severe EMG abnormalities, although there was substantial overlap between groups, indicating that there may be little or no true difference. The results for CNFD, fractal dimension and tortuosity are presented in ESM Figs 1–3. The results for CNFD, fractal dimension and tortuosity were consistent across the various degrees of EMG abnormalities, showing no clear differences within either the group with neuropathic pain or the group without pain. For CNFD, individuals with neuropathic pain and prediabetes had higher values than individuals without neuropathic pain, and those with neuropathic pain and type 2 diabetes had lower values; however, these differences were not statistically significant.

### Associations of EMG with WF-CCM

As shown in Table 2, EMG abnormalities, based on the general composite score (nerve conduction velocities, distal motor latencies and amplitudes), were associated with lower corneal nerve parameters, and a stronger association was seen for severe EMG abnormalities, but most of the associations were not statistically significant, with the exception of fractal dimension in individuals with severe EMG abnormalities. However, when using the axonal degeneration composite score based on the CMAP amplitudes of the tibial/peroneal nerves and SNAP amplitudes of the sural nerve, regression analyses revealed statistically significant associations with WF-CCM parameters (CNFL: β=−0.51 [95% CI −0.78, −0.24]; CNFD: β=−1.56 [95% CI −3.04, −0.08]; CNBD: β=−3.08 [95% CI −5.51, −0.64]; fractal dimension −0.01 [95% CI −0.02, −0.01]; tortuosity −0.05 [95% CI −0.08, −0.01]; model crude; all *p*<0.05), after adjustment statistically significant associations persisted only for CNFL, fractal dimension and tortuosity (Table 3).

**Table 2:** Associations of EMG abnormalities based on the general composite score with corneal nerve parameters in the total population

Model	CNFL (mm/mm^2^)	CNFD (fibres/mm^2^)	CNBD (branches/mm^2^)	Corneal nerve fractal dimension	Tortuosity
Mild EMG abnormalities vs no EMG abnormalities					
Crude model	−0.28 (−0.67, 0.11)	−0.37 (−2.54, 1.80)	−0.80 (−4.37, 2.78)	−0.01 (−0.01, 0.00)	−0.03 (−0.08, 0.016)
Model 1	−0.19 (−0.58, 0.20)	−0.09 (−2.25, 2.08)	−0.23 (−3.79, 3.32)	0.00 (−0.01, 0.00)	−0.02 (−0.07, 0.03)
Model 2	−0.05 (−0.45, 0.35)	0.63 (−1.63, 2.89)	0.72 (−3.01, 4.44)	0.00 (−0.01, 0.01)	−0.02 (−0.07, 0.03)
Moderate EMG abnormalities vs no EMG abnormalities					
Crude model	−0.82 (−1.88, 0.23)	−3.84 (−9.66, 1.98)	−5.58 (−15.16, 4.00)	−0.02 (−0.04, 0.01)	0.01 (−0.12, 0.14)
Model 1	−0.83 (−1.87, 0.22)	−3.85 (−9.64, 1.95)	−5.60 (−15.12, 3.93)	−0.02 (−0.04, 0.01)	0.01 (−0.11, 0.14)
Model 2	−0.39 (−1.47, 0.69)	−2.05 (−8.10, 3.99)	−3.33 (−13.29, 6.63)	−0.01 (−0.03, 0.02)	0.03 (−0.10, 0.16)
Severe EMG abnormalities vs no EMG abnormalities					
Crude model	−1.42 (−3.41, 0.57)	−9.45 (−20.44, 1.54)	−14.54 (−32.63, 3.55)	−0.05 (−0.09, −0.01)*	0.03 (−0.21, 0.27)
Model 1	−1.52 (−3.48, 0.45)	−9.75 (−20.70, 1.19)	−15.15 (−33.14, 2.84)	−0.05 (−0.09, −0.01)*	0.02 (−0.22, 0.25)
Model 2	−1.23 (−3.51, 1.05)	−7.54 (−20.32, 5.24)	−12.29 (−33.34, 8.77)	−0.05 (−0.10, −0.00)*	−0.06 (−0.34, 0.22)

Values are unstandardised β (95% CI)

Multivariable linear regression was performed for CNFL, CNFD, CNBD, fractal dimension and tortuosity. Model 1 was adjusted for CCM lag time; model 2 was adjusted for CCM lag time, age, sex, BMI, height and skin temperature

Asterisks indicate values that are statistically significant (*p<0.05)

**Table 3:** Associations of EMG abnormalities based on the axonal degeneration composite score with corneal nerve parameters in the total population

Model	CNFL (mm/mm^2^)	CNFD (fibres/mm^2^)	CNBD (branches/mm^2^)	Corneal nerve fractal dimension	Tortuosity
Crude model	−0.51 (−0.78, −0.24)***	−1.56 (−3.04, −0.08)*	−3.08 (−5.51, −0.64)*	−0.01 (−0.02, −0.01)***	−0.05 (−0.08, −0.01)**
Model 1	−0.53 (−0.79, −0.27)***	−1.63 (−3.10, −0.15)*	−3.21 (−5.63, −0.79)**	−0.02 (−0.02, −0.01)***	−0.05 (−0.08, −0.02)**
Model 2	−0.40 (−0.68, −0.12)**	−0.95 (−2.51, 0.62)	−2.35 (−4.93, 0.23)	−0.01 (−0.01, 0.00)**	−0.05 (−0.08, −0.02)**

Values are unstandardised β (95% CI)

Model 1 was adjusted for WF-CCM lag time; model 2 was adjusted for WF-CCM lag time, age, sex, BMI, height and skin temperature

Asterisks indicate values that are statistically significant (*p<0.05, **p<0.01, ***p<0.001)

Subgroup analyses revealed negative associations between EMG abnormalities and corneal nerve parameters in individuals with type 2 diabetes. In contrast, participants with normal glucose metabolism showed positive associations between certain WF-CCM parameters and EMG abnormalities. Prediabetes was generally characterised by predominantly positive associations; however, this group exhibited the weakest associations and the widest CI widths (ESM Tables 7–9). After adjustment for confounding factors, the strength of association increased for some WF-CCM parameters, but decreased for others.

### Associations of neuropathic pain with WF-CCM

As shown in Table 4, neuropathic pain showed no statistically significant associations with corneal nerve parameters for the entire group (CNFL: −0.06 [95% CI −0.57, 0.45]; CNFD: −1.15 [95% CI −4.00, 1.71]; CNBD: −0.22 [95% CI −4.92, 4.48]; fractal dimension: 0.00 [95% CI −0.01, 0.01]; tortuosity: 0.02 [95% CI −0.04, 0.08]; model 2). Subgroup analysis showed similar results, although stronger associations were observed for CNBD for both prediabetes and type 2 diabetes mellitus. The strength of these associations decreased after adjusting for the degree of EMG abnormality (model 3). Statistically significant positive associations between neuropathic pain and tortuosity were present in individuals with prediabetes (*p*<0.01).

**Table 4:** Associations of neuropathic pain with corneal nerve fibre parameters

Model	CNFL (mm/mm^2^)	CNFD (fibres/mm^2^)	CNBD (branches/mm^2^)	Corneal nerve fractal dimension	Tortuosity
Total					
Crude model	−0.19 (−0.71, 0.33)	−1.35 (−4.21, 1.52)	−0.54 (−5.24, 4.17)	0.00 (−0.01, 0.01)	0.01 (−0.05, 0.07)
Model 1	−0.18 (−0.69, 0.33)	−1.32 (−4.17, 1.53)	−0.49 (−5.17, 4.19)	0.00 (−0.01, 0.01)	0.01 (−0.05, 0.07)
Model 2	−0.06 (−0.57, 0.45)	−1.15 (−4.00, 1.71)	−0.22 (−4.92, 4.48)	0.00 (−0.01, 0.01)	0.02 (−0.04, 0.08)
Model 3	0.06 (−0.46, 0.59)	−0.84 (−3.79, 2.11)	0.79 (−4.07, 5.65)	0.00 (−0.01, 0.02)	0.04 (−0.03, 0.10)
Normal glucose metabolism					
Crude model	−0.21 (−0.95, 0.52)	−0.13 (−4.17, 3.92)	0.02 (−6.70, 6.74)	0.00 (−0.02, 0.01)	−0.08 (−0.17, 0.01)
Model 1	−0.22 (−0.95, 0.51)	−0.14 (−4.18, 3.90)	−0.01 (−6.72, 6.70)	0.00 (−0.02, 0.01)	−0.08 (−0.17, 0.01)
Model 2	−0.21 (−0.94, 0.52)	−0.42 (−4.46, 3.63)	−0.42 (−7.15, 6.31)	0.00 (−0.02, 0.01)	−0.07 (−0.16, 0.03)
Model 3	−0.02 (−0.77, 0.73)	−0.11 (−4.28, 4.05)	0.77 (−6.16, 7.70)	0.00 (−0.02, 0.01)	−0.04 (−0.13, 0.06)
Prediabetes					
Crude model	0.67 (−0.65, 1.99)	−0.47 (−7.97, 7.04)	1.28 (−10.68, 13.24)	0.02 (−0.01, 0.05)	0.24 (0.09, 0.39)**
Model 1	0.64 (−0.65, 1.93)	−0.58 (−8.01, 6.84)	1.06 (−10.72, 12.85)	0.02 (−0.01, 0.05)	0.24 (0.09, 0.39)**
Model 2	0.71 (−0.57, 1.98)	−0.36 (−7.74, 7.03)	1.51 (−10.25, 13.27)	0.02 (0.00, 0.05)	0.24 (0.09, 0.39)**
Model 3	0.70 (−0.63, 2.04)	0.00 (−7.79, 7.79)	1.99 (−10.43, 14.40)	0.02 (0.00, 0.05)	0.23 (0.07, 0.39)**
Type 2 diabetes					
Crude model	0.05 (−0.86, 0.96)	−1.10 (−6.09, 3.90)	1.06 (−7.14, 9.26)	0.01 (−0.01, 0.03)	0.07 (−0.04, 0.17)
Model 1	−0.05 (−0.93, 0.84)	−1.46 (−6.39, 3.47)	0.42 (−7.66, 8.50)	0.01 (−0.02, 0.03)	0.05 (−0.05, 0.16)
Model 2	−0.03 (−0.91, 0.86)	−1.60 (−6.52, 3.32)	0.23 (−7.84, 8.31)	0.01 (−0.01, 0.03)	0.06 (−0.04, 0.16)
Model 3	0.04 (−0.88, 0.95)	−1.33 (−6.46, 3.80)	1.24 (−7.19, 9.67)	0.01 (−0.01, 0.03)	0.06 (−0.05, 0.17)

Values are unstandardised β (95% CI)

Multivariable linear regression was performed for CNFL, CNFD, CNBD, fractal dimension and tortuosity. Model 1 was adjusted for CCM lag time; model 2 was adjusted for CCM lag time, age, sex, BMI and height; model 3 was adjusted for CCM lag time, age, sex, BMI, height, skin temperature and EMG score

Asterisks indicate statistical significance (**p<0.01)

### Sensitivity analysis

No significant interactions were detected for diabetes status or sex (ESM Figs 4 and 5). Sensitivity analyses in which participants with characteristics that could influence the results were excluded yielded findings that were consistent with the main analyses, as described in ESM Results. In contrast, additional sensitivity analyses focusing on axonal degeneration (using the composite score and continuous measures of tibial and sural nerve amplitude) revealed statistically significant negative associations with WF-CCM parameters (ESM Tables 10–12). Adjustment for neuropathic pain medication use did not change the main findings (ESM Table 13). Performing sensitivity analyses that excluded outliers (ESM Tables 14 and 15) weakened the estimates but did not change the direction or significance of associations.

## Discussion

In this large population-based study, we investigated the associations between EMG, neuropathic pain and WF-CCM parameters across categories of glucose metabolism (normal, prediabetes and type 2 diabetes). The study found associations between the axonal degeneration composite score and WF-CCM parameters, but no associations were observed between the more general EMG composite score and WF-CCM parameters. In contrast, the results show that there was no significant association between neuropathic pain and corneal nerve parameters.

The absence of an association between the general EMG composite score, based on both nerve conduction velocity, distal latencies and amplitudes and WF-CCM variables, may not be entirely unexpected, as EMG primarily assesses large-fibre function, while CCM assesses small-fibre structure. However, as large-fibre involvement typically reflects more advanced stages of neuropathy, some degree of association may have been anticipated. Interestingly, when we repeated our analyses using only nerve conduction amplitudes (peroneal, tibial and sural) as markers of axonal degeneration, we observed significant associations between the axonal degeneration composite score and WF-CCM parameters, which remained after adjustment in the fully adjusted model (model 2), except for CNFD and CNBD. As polyneuropathy is primarily characterised by axonal degeneration in the early stages, use of a composite score that is focused specifically on axonal loss may better capture the underlying pathology than one incorporating a broader range of EMG measures. Notably, focusing solely on the sensory sural nerve amplitude did not yield a stronger association with WF-CCM parameters compared with the composite score that included sensory and motor nerve conduction amplitudes.

Consistent with our observation of axonal degeneration in lower-leg nerves being associated with multiple WF-CCM parameters in individuals with and without prediabetes and diabetes, there is an increasing body of evidence supporting associations between DPN and CCM abnormalities [18, 19]. These associations have been found in prediabetes and type 1 and type 2 diabetes [17]. Mokhtar et al reported linear associations between WF-CCM parameters and measures of glycaemic control in the Maastricht Study [14], and as hyperglycaemia, dyslipidaemia and inflammation drive microvascular impairment, which correlates with neuronal loss through endoneurial microangiopathic changes, it may be speculated that this association is due to the development of polyneuropathy [3, 14, 18, 31, 32]. However, our findings revealed no significant association between EMG composite score and WF-CCM parameters across glucose metabolism groups, suggesting that the results reported by Mokhtar et al cannot be attributed solely to large-fibre polyneuropathy. We found no significant associations between neuropathic pain and CNFL, CNFD or CNBD. The literature reports similar findings [22, 33, 34], although associations have been described by some. Kalteniece et al found that all CCM parameters were reduced in individuals with painful DPN, including the inferior whorl nerve length, and described associations between CCM parameters and the severity of pain [35, 36]. while Püttgen et al found enhanced CNBD in individuals with painful DPN and proposed that the nerve regeneration was not able to compensate for the overall nerve loss in DPN [21]. Regeneration of damaged nerves is thought to be involved in the development of neuropathic pain, and previous studies have reported increased CNBD in painful DPN and described tortuosity as a morphological marker of nerve regeneration [21, 37]; however, the present study did not indicate an association between these parameters and neuropathic pain. Sierra-Silvestre et al observed an increased presence and frequency of axonal swelling and microneuromas, which are a sign of damaged nerves and again are thought to play a role in the pathophysiology of neuropathic pain [34]. Evaluation of other measures such as microneuromas in individuals with neuropathic pain may be of interest to investigate the pathophysiology beyond CCM parameters (CNFL, CNFD, CNBD, fractal dimension and tortuosity) [34, 38].

Patient-reported questionnaires have been proposed as a method for stratifying painful neuropathy into sensory phenotypes, which may be related to underlying mechanisms [39, 40]. We performed a pre-planned exploratory analysis to test whether CNFL, CNBD, CNFD, fractal dimension and tortuosity are more closely associated with specific pain types (electric, cold, burning and mixed pain) defined by DN4 responses, but found no associations between pain types and corneal nerve parameters.

This study has several limitations that should be considered. First, the cross-sectional design limits our ability to draw conclusions about causality between neuropathic pain and corneal nerve parameters. However, a 7-year longitudinal follow-up study is currently underway to address this limitation. Second, neuropathic pain was assessed using the DN4 questionnaire, which, while widely used, is a screening tool and may not capture the full complexity or intensity of neuropathic pain diagnosis, potentially leading to underestimation of associations with corneal nerve parameters. Additionally, neuropathy was evaluated using EMG, which primarily assesses large-fibre function, whereas our primary outcome, WF-CCM, reflects small-fibre integrity. This discrepancy may contribute to the modest associations observed. Third, despite the overall large sample size, the number of individuals with diabetes and with severe neuropathy or pain symptoms was relatively small. This may partly reflect a well-managed cohort with relatively short diabetes duration, potentially limiting the generalisability of our findings to populations with more advanced disease. Lastly, the cohort reflects the limited racial and ethnic diversity of the region, with 98.8% of participants self-identifying as White [24].

Biological sex may influence both peripheral nerve pathology and pain perception [41]. The study population was evenly distributed by sex (52% female), and sex was included as a covariate in regression models; no interactions were observed between sex and exposures (EMG abnormalities and neuropathic pain). Although the study was not powered for sex-specific analyses, the findings are probably generalisable across biological sex.

The present study uses WF-CCM, which integrates real-time wide-field mapping of composite imaging, thereby removing the image selection process. Together with fully automated analysis of the images, use of this method is thought to decrease analysis time and improve reliability [25, 26], making CCM more readily translatable into clinical practice. It should be noted that the composite images include the peripheral cornea and therefore produce different results than previously published protocols focusing on central corneal changes [42].

In conclusion, WF-CCM allows fast non-invasive evaluation of the pathophysiology in neuropathy. While this study found no significant association between a general EMG composite score and WF-CCM parameters, apart from fractal dimension in individuals with severe EMG abnormalities, significant associations were observed between the more specific axonal degeneration composite score and CNFL, fractal dimension and tortuosity. No associations were found between neuropathic pain and CCM parameters.

## Supplementary Information

Below is the link to the electronic supplementary material.

Supplementary file1 (PDF 905 KB)

## Data Availability

The data from this study derive from the Maastricht Study, but restrictions apply to the availability of these data, which were used under license for the current study. However, data are available from the authors on reasonable request and with permission from the Maastricht Study management team. Data analysis is available from the authors on reasonable request.

## Abbreviations

CCM: Corneal confocal microscopy
CMAP: Compound muscle action potential
CNBD: Corneal nerve branch density
CNFD: Corneal nerve fibre density
CNFL: Corneal nerve fibre length
DN4: Modified Douleur Neuropathique en 4 Questions
DPN: Diabetic polyneuropathy
EMG: Electromyography
SNAP: Sensory nerve action potential
WF-CCM: Wide-field corneal confocal microscopy
