# Supplementary material for: Association of corneal nerve parameters with nerve abnormalities and neuropathic pain in prediabetes and type 2 diabetes: the Maastricht Study
Source: Diabetologia. 2026 Feb 10;69(6):1519–31. doi: 10.1007/s00125-026-06676-8 (PMC13109147; doi:10.1007/s00125-026-06676-8)

Electronic supplementary material (ESM)

Association of corneal nerve parameters with nerve abnormalities and neuropathic pain in prediabetes and type 2 diabetes: the Maastricht Study

Mette Krabsmark Borbjerg, Sara Mokhtar, Nadia Sutedja, Annemarie Koster, Carsten Dahl Mørch, Tos T. J. M. Berendschot, Nicolaas Schaper, Niels Ejskjaer, Johan Røikjer

Methods

Data analysis

Population characteristics were described for the total population (*N*=9187) grouped by eligibility status. In the main text population characteristics were described by neuropathic pain for the total population, in the **ESM Results**, population characteristics by neuropathic pain status are presented separately for individuals with normal glucose metabolism, prediabetes, and type 2 diabetes using appropriate descriptive statistics.

Normality of the data were assessed using QQ-plot and density plot. As the corneal parameters were not normally distributed within the subgroups defined by nerve conduction score and neuropathic pain status, we assessed statistical differences using the non-parametric Mann Whitney U-test. Comparisons were conducted within each level of nerve conduction abnormality (no, mild, moderate, or severe), comparing participants with and without neuropathic pain. Significant p-values are reported in the figures.

Regression stratified by glucose metabolism status

To examine the association between determinants (EMG abnormality or neuropathic pain) and corneal nerve parameters across glucose metabolism status linear regression analysis was performed as a subgroup analysis performed stratified based on glucose metabolism status. All associations are expressed as unstandardized regression coefficients (β) with corresponding 95% CI. Data precision is expressed as width of the confidence interval.

Nerve function Associations of determinants (i.e. degree of nerve abnormalities [none, mild, moderate, or severe EMG abnormalities]) with outcomes (i.e., CNFD, CNFL, CNBD, corneal nerve fractal dimension, and tortuosity) were assessed using multivariable linear regression. For outcomes with non-normally distributed residuals, a sensitivity analysis removing outliers were performed. The crude model examined EMG abnormality and corneal nerve parameters. Model

1 was adjusted for corneal confocal microscopy lag time. Model 2, in addition to the adjustment in model 1, was adjusted for age, sex, BMI, height, and skin temperature. We chose these variables because they are key potential confounders.

Neuropathic pain Associations of determinants (i.e. presence of neuropathic pain (i.e. and neuropathic pain [yes/no]) with outcomes (i.e., CNFD, CNFL, CNBD, corneal nerve fractal dimension, and tortuosity) were assessed using multivariable linear regression. For outcomes with non-normally distributed residuals, a sensitivity analysis removing outliers were performed. The crude model examined neuropathic pain and corneal nerve parameters. Model 1 analysis was adjusted for corneal confocal microscopy lag time. Model 2, in addition to the adjustment in the model 1, was adjusted for age, sex, BMI, and height. Model 3, in addition to the adjustments in model 2, was adjusted for degree of nerve abnormalities [none, mild, moderate, or severe EMG abnormalities] and skin temperature.

Interaction analysis

Interaction analysis was performed for diabetes status based on the design of the Maastricht study which is characterized by an alternative recruitment for individuals with type 2 diabetes, while interaction analysis was performed for sex based on international consensuses.

Sensitivity analysis

Selected sensitivity analyses are presented below. Additionally, the regression analysis was performed after excluding participants with factors that could potentially influence the outcome (high alcohol intake n = 737, history of cancer n = 185, and eGFR < 30 n = 8) and excluding individuals with catch-up visit or diagnosis of type 2 diabetes < 1 year (n = 174). These exclusions did not substantially alter the results of the regression analyses, and the results are not shown.

Regression analysis - Nerve Conduction Studies

In the main analysis we used a composite score that included all relevant EMG results in a composite score. Including data of various and different aspects of peripheral nerve function into one variable can be difficult to interpret and might result is false negative conclusions. We therefore planned to perform three sensitivity analysis focusing more specifically on axonal degeneration, as this is a key pathological feature in large fibre axonal polyneuropathy.

Sensitivity analysis 1: Axonal degeneration composite score

A composite score was calculated including the following measures: CMAP amplitude of the peroneal and tibial nerves, along with SNAP amplitude of the sural nerve. Nerve measures were categorised as follows: 0 indicating normal, 1 representing mild abnormalities (10th percentile), 2 for moderate abnormalities (5th percentile), and 3 indicating severe abnormalities (2.5^{th} percentile). The axonal degeneration composite score was then derived by summing these scores and dividing by the number of measures and included in the regression analysis as the primary predictor.

- Crude model: Axonal degeneration
- Model 1: adjusted for corneal confocal microscopy lag time.
- Model 2, adjusted for corneal confocal microscopy lag time, age, sex, BMI, height, and skin temperature.

Sensitivity analysis 2: Sural nerve amplitude (continuous measures)

SNAP amplitude of the sural nerve was included in the analysis as the primary predictor.

- Crude model: Sural nerve amplitude (μV)
- Model 1: adjusted for corneal confocal microscopy lag time.
- Model 2, adjusted for corneal confocal microscopy lag time, age, sex, BMI, height, and skin temperature.

Sensitivity analysis 3: Tibial nerve amplitude (continuous measures)

CMAP amplitude of the tibial nerve was included in the analysis as the primary predictor.

- Crude model: Tibial nerve amplitude (μV)
- Model 1: adjusted for corneal confocal microscopy lag time.
- Model 2, adjusted for corneal confocal microscopy lag time, age, sex, BMI, height, and skin temperature.

Regression analysis – Pain medication

Given that the primary analysis focuses on pain, the use of pain medication could confound the results. Therefore, a sensitivity analysis was conducted to assess the impact of pain medication use.

Sensitivity analysis: Pain medication

A binary variable, *pain_med*, was created to indicate the use of neuropathic pain medication. Individuals were classified as 'yes' if they reported using any of the specified medications, and 'no' if none were used. The list of medication included: pregabalin, gabapentin, carbamazepine, amitriptyline, nortriptyline, duloxetine.

- Crude model: Neuropathic pain
- Model 1: adjusted for corneal confocal microscopy lag time.
- Model 2, adjusted for corneal confocal microscopy lag time, age, sex, BMI, and height.
- Model 3, adjusted for corneal confocal microscopy lag time, age, sex, BMI, and height, degree of nerve abnormalities [none, mild, moderate, or severe EMG abnormalities], skin temperature and **pain_med**.

Regression analysis – Outliers

A sensitivity analysis was conducted to assess the robustness of the results by addressing potential outliers in the data. This was prompted by the observation of non-normally distributed residuals in the primary linear regression models assessing associations between EMG parameters or neuropathic pain and WF-CCM outcomes. Log-transformation of the outcome variables (CNBD and fractal dimension) did not adequately normalize the residuals. However, as no evidence of heteroskedasticity was found, linear regression was retained for the main analysis.

To evaluate the influence of extreme values, influential observations were identified based on standardized residuals (absolute value >2) and excluded in a sensitivity analysis. This was performed for both the EMG and neuropathic pain models.

EMG composite score

- Crude model: EMG

- Model 1: adjusted for corneal confocal microscopy lag time.
- Model 2, adjusted for corneal confocal microscopy lag time, age, sex, BMI, height, and skin temperature.

Neuropathic pain

- Crude model: Neuropathic pain
- Model 1: adjusted for corneal confocal microscopy lag time.
- Model 2, adjusted for corneal confocal microscopy lag time, age, sex, BMI, and height.
- Model 3, adjusted for corneal confocal microscopy lag time, age, sex, BMI, and height, degree of nerve abnormalities [none, mild, moderate, or severe EMG abnormalities], and skin temperature.

Results

The Maastricht Study cohort consists of 9187 participants. Of these, 3425 individuals were included in the present analyses. **Figure 1** provides an overview of the selection process, and **ESM Table 1** presents the baseline demographic and clinical characteristics of participants included in the analyses compared with those excluded.

Population characteristics stratified by neuropathic pain status within each glucose metabolism group (normal glucose metabolism, prediabetes, and type 2 diabetes) are presented in **ESM Tables 2–4**.

For the total population (*n* = 3,425), **ESM Table 5** summarises the distribution of EMG abnormalities, mean sural and tibial amplitudes, and corneal nerve measures according to neuropathic pain status. **ESM Table 6** presents sural and tibial amplitudes and corneal nerve parameters stratified by the degree of EMG abnormality.

CNFD, fractal dimension, and tortuosity in relation to neuropathic pain, stratified by degree of EMG abnormality and glucose metabolism status, are presented in **ESM Fig 1-3**. The results for CNFD, fractal dimension and tortuosity were consistent across the different degrees of EMG abnormalities, showing no clear differences within either the group with neuropathic pain or the group without pain. For CNFD, compared with individuals without neuropathic pain, individuals

with neuropathic pain and prediabetes had higher values and those with neuropathic pain and type 2 diabetes had lower values; however, these differences were not statistically significant.

Regression stratified by glucose metabolism status

Subgroup analyses revealed negative associations between EMG abnormalities and corneal nerve parameters in individuals with type 2 diabetes. In contrast, participants with normal glucose metabolism showed positive associations between certain WF-CCM parameters (CNFD, CNBD) and mild EMG abnormalities, while moderate and severe EMG abnormalities showed predominantly negative associations. Prediabetes was generally characterized by predominantly positive associations, with weak associations and wide CI widths, see **ESM Tables 7-9**. After adjustment for confounding factors, the strength of association increased for some WF-CCM parameters, while it decreased for others.

Interaction analysis

Interaction analyses showed no significant interactions between diabetes status and the exposures (EMG abnormalities and neuropathic pain) or between sex and these exposures. Results are presented in **ESM Fig 4-5**.

Sensitivity analysis

Regression analysis - Nerve Conduction Studies

<u>Sensitivity analysis 1: Axonal degeneration composite score</u>

Results are presented in **ESM Table 10**. In summary, we observed significant negative associations between axonal damage and all CCM parameters in the crude model and model 1. After adjusting for potential confounders, including age, sex, BMI, height and skin temperature in Model 2, statistically significant negative associations were observed for CNFL, CNBD, and tortuosity.

<u>Sensitivity analysis 2: Sural nerve amplitude (continuous measures)</u>

Results are presented in **ESM Table 11**. In summary, we observed significant positive associations between sural nerve amplitude and CNFL, fractal dimension, and tortuosity in the crude model. In model 1 associations were observed for CNFL, CNFD, fractal dimension, and

tortuosity. After adjusting for potential confounders, including age, sex, BMI, height and skin temperature in Model 2, statistically significant positive associations were observed for only tortuosity.

Sensitivity analysis 3: Tibial nerve amplitude (continuous measures)

Results are presented in **ESM Table 12**. In summary, we observed significant positive associations between tibial nerve amplitude and CNFL, CNFD, CNBD, and fractal dimension in the crude model and model 1. After adjusting for potential confounders, including age, sex, BMI, height and skin temperature in Model 2, no statistically significant associations were observed.

Regression analysis – Pain medication

When pain medication use was added to the fully adjusted model, the associations between neuropathic pain and corneal nerve parameters showed no meaningful changes, with effect estimates remaining close to those observed in Model 2. Results are presented in **ESM Table 13**.

Regression analysis – Outliers

Results of the sensitivity analysis assessing the influence of outliers are presented in **ESM Tables 14–15**. The associations between severe EMG abnormalities and WF-CCM parameters are weaker. Though, the direction and significance of the associations remained largely unchanged, supporting the robustness of the main findings.

Tables

ESM Table 1 Complete study population – characteristics

Variable	Total (N=9187)	Excluded (N=5762)	Included (N=3425)	p-value
Age (years)	59.5 ± 8.8	59.7 ± 8.8	59.2 ± 8.7	0.004
Female sex [a]	4616 (50.2)	2847 (49.4)	1769 (51.6)	0.040
Race and ethnicity: White (%)[a]	9051 (98.5)	5668 (98.4)	3383 (98.8)	0.200
Fasting plasma glucose (mmol/l)	5.4 (5.0, 6.1)	5.4 (5.0, 6.2)	5.3 (4.9, 6.0)	< 0.001
HbA_{1c} (%)	5.5 (5.3, 5.9)	5.5 (5.3, 6.0)	5.4 (5.2, 5.9)	< 0.001
HbA_{1c} (mmol/mol)	37.0 (34.0, 41.0)	37.0 (34.0, 42.0)	36.0 (33.0, 41.0)	< 0.001
Glucose metabolism				< 0.001
Normal Glucose Metabolism	5747 (62.6)	3489 (60.6)	2258 (65.9)	
Prediabetes	1382 (15.0)	868 (15.1)	514 (15.0)	
Type 2 Diabetes	2005 (21.8)	1352 (23.5)	653 (19.1)	
Diabetes Duration (years)	4.0 (0, 9.0)	4.0 (0, 10.0)	3.0 (0, 8.0)	< 0.001
Taking diabetes medication	1492 (16.2)	1047 (18.2)	445 (13.0)	< 0.001
BMI (kg/m^2)	26.9 ± 4.5	27.0 ± 4.6	26.7 ± 4.3	< 0.001
Waist circumference(cm)	95.0 ± 13.6	95.5 ± 13.9	94.1 ± 13.1	< 0.001
Office systolic blood pressure (mmHg)	133 ± 17.9	134 ± 18.2	133 ± 17.6	0.008
Office diastolic blood pressure (mmHg)	75.4 ± 9.9	75.4 ± 9.9	75.4 ± 9.9	0.886
Taking antihypertensive medication	3300 (35.9)	2140 (37.1)	1160 (33.9)	< 0.001
Serum cholesterol HDL-cholesterol (mmol/l)	1.5 ± 0.5	1.5 ± 0.5	1.6 ± 0.5	0.002
Serum cholesterol LDL-cholesterol (mmol/l)	3.1 ± 1.0	3.1 ± 1.0	3.1 ± 1.0	0.960
Serum total cholesterol (mmol/l)	5.2 ± 1.1	5.2 ± 1.1	5.2 ± 1.1	0.145
eGFR (ml/min per 1.73m^2) [b]	89.3 (79.0, 98.8)	89.5 (79.0, 99.1)	88.8 (79.2, 97.9)	0.451
Smoking categories				0.280
Current	1201 (13.1)	774 (13.4)	427 (12.5)	
Former	4412 (48.0)	2749 (47.7)	1663 (48.6)	
Never	3503 (38.1)	2168 (37.6)	1335 (39.0)	
Alcohol consumption status[c]				0.139
High	2036 (22.2)	1299 (22.5)	737 (21.5)	
Low	5384 (58.6)	3316 (57.5)	2068 (60.4)	
None	1692 (18.4)	1073 (18.6)	619 (18.1)	

Demographics and clinical characteristics for all participants from the Maastricht study, stratified by inclusion status in the current study. Data are *n* (%), mean ± SD or median (IQR) as appropriate based on variable type and distribution.

Neuropathic pain was defined as a DN4 interview score ≥3

[a]Sex and race/ethnicity were self-reported by participants at enrolment.

[b]eGFR was calculated using the CKD-EPI equations (with both serum creatinine and cystatin C)

[c]High alcohol consumption was defined as >7 units/week for women and >14 units/week for men

ESM Table 2 Normal glucose metabolism - study population characteristics

Variable	Total (N=2258)	Neuropathic pain (N=145)	No neuropathic pain (N=2113)
Age (years)	57.6 ± 8.6	59.4 ± 8.4	57.5 ± 8.6
Female sex [a]	1301 (57.6)	99 (68.3)	1202 (56.9)
Fasting plasma glucose (mmol/mol)	5.1 (4.8, 5.4)	5.2 (4.9, 5.5)	5.1 (4.8, 5.4)
HbA_{1c} (%)	5.4 (5.1, 5.5)	5.4 (5.2, 5.6)	5.4 (5.1, 5.5)
HbA_{1c} (mmol/mol)	35.0 (32.0, 37.0)	36.0 (33.0, 38.0)	35.0 (32.0, 37.0)
BMI (kg/m^2)	27.8 ± 4.3	28.7 ± 4.0	27.7 ± 4.3
Waist circumference(cm)	90.0 ± 11.3	90.6 ± 12.7	90.0 ± 11.2
Office systolic blood pressure (mmHg)	129 ± 16.7	130 ± 16.2	129 ± 16.8
Office diastolic blood pressure (mmHg)	74.6 ± 9.8	74.2 ± 9.5	74.6 ± 9.9
Taking antihypertensive medication	488 (21.6)	50 (34.5)	438 (20.7)
Serum cholesterol HDL-cholesterol (mmol/l)	1.7 ± 0.5	1.6 ± 0.5	1.7 ± 0.5
Serum cholesterol LDL-cholesterol (mmol/l)	3.2 ± 0.9	3.3 ± 0.9	3.2 ± 0.9
Serum total cholesterol (mmol/l)	5.4 ± 1.0	5.5 ± 1.0	5.4 ± 1.0
Smoking categories			
Current	278 (12.3)	32 (22.1)	246 (11.6)
Former	1027 (45.5)	68 (46.9)	959 (45.4)
Never	953 (42.2)	45 (31.0)	908 (43.0)
Alcohol consumption status[c]			
High	495 (21.9)	30 (20.7)	465 (22.0)
Low	1389 (61.5)	82 (56.6)	1307 (61.9)
None	373 (16.5)	33 (22.8)	340 (16.1)
EMG composite score			
0, No abnormality	1886 (83.5)	1772 (83.9)	114 (78.6)
1, Mild abnormality	342 (15.1)	313 (14.8)	29 (20.0)
2, Moderate abnormality	24 (1.1)	22 (1.0)	2 (1.4)
3, Severe abnormality	6 (0.3)	6 (0.3)	0 (0)

Demographics and clinical characteristics for all participants with normal glucose metabolism, stratified by presence of neuropathic pain. Data are *n* (%), mean ± SD or median (IQR) as appropriate based on variable type and distribution.

Neuropathic pain was defined as a DN4 interview score ≥3

[a]Sex was self-reported by participants at enrolment.

[b]eGFR was calculated using the CKD-EPI equations (with both serum creatinine and cystatin C)

[c]High alcohol consumption was defined as >7 units/week for women and >14 units/week for men

ESM Table 3 Prediabetes - study population characteristics

Variable	Total (N=514)	Neuropathic pain (N=48)	No neuropathic pain (N=466)
Age (years)	62.0 ± 8.4	62.2 ± 7.9	61.9 ± 8.5
Female sex [a]	244 (47.5)	26 (54.2)	218 (46.8)
Fasting plasma glucose (mmol/mol)	5.9 (5.4, 6.30)	5.9 (5.5, 6.2)	5.9 (5.4, 6.3)
HbA_{1c} (%)	5.6 (5.4, 5.9)	5.7 (5.5, 5.9)	5.5 (5.4, 5.9)
HbA_{1c} (mmol/mol)	38.0 (35.0, 41.0)	38.5 (36.8, 41.0)	37.0 (35.0, 41.0)
BMI (kg/m^2)	27.8 ± 4.3	28.7 ± 4.0	27.7 ± 4.3
Waist circumference(cm)	98.1 ± 12.4	101 ± 11.2	97.8 ± 12.5
Office systolic blood pressure (mmHg)	137 ± 17.3	135 ± 19.1	137 ± 17.1
Office diastolic blood pressure (mmHg)	76.6 ± 9.8	76.6 ± 10.8	76.6 ± 9.8
Taking antihypertensive medication	231 (44.9)	30 (62.5)	201 (43.1)
Serum cholesterol HDL-cholesterol (mmol/l)	1.5 ± 0.4	1.4 ± 0.4	1.5 ± 0.4
Serum cholesterol LDL-cholesterol (mmol/l)	3.2 ± 1.0	3.0 ± 0.9	3.2 ± 1.0
Serum total cholesterol (mmol/l)	5.3 ± 1.1	5.2 ± 1.1	5.4 ± 1.1
Smoking categories			
Current	59 (11.5)	8 (16.7)	51 (10.9)
Former	279 (54.3)	30 (62.5)	249 (53.4)
Never	176 (34.2)	10 (20.8)	166 (35.6)
Alcohol consumption status[c]			
High	124 (24.1)	9 (18.8)	115 (24.7)
Low	306 (59.5)	26 (54.2)	280 (60.1)
None	84 (16.3)	13 (27.1)	71 (15.2)
EMG composite score			
0, No abnormality	409 (79.6)	376 (80.7)	33 (68.8)
1, Mild abnormality	87 (16.9)	78 (16.7)	9 (18.8)
2, Moderate abnormality	13 (2.5)	10 (2.1)	3 (6.3)
3, Severe abnormality	5 (1.0)	2 (0.4)	3 (6.3)

Demographics and clinical characteristics for all participants with prediabetes, stratified by presence of neuropathic pain. Data are *n* (%), mean ± SD or median (IQR) as appropriate based on variable type and distribution.

Neuropathic pain was defined as a DN4 interview score ≥3

[a]Sex was self-reported by participants at enrolment.

[b]eGFR was calculated using the CKD-EPI equations (with both serum creatinine and cystatin C)

[c]High alcohol consumption was defined as >7 units/week for women and >14 units/week for men

ESM Table 4 Diabetes mellitus type 2 - study population characteristics

Variable	Total (N=653)	Neuropathic pain (N=113)	No neuropathic pain (N=540)
Age (years)	62.6 ± 7.9	62.6 ± 7.6	62.6 ± 8.0
Female sex [a]	224 (34.3)	44 (38.9)	180 (33.3)
Fasting plasma glucose (mmol/mol)	7.3 (6.5, 8.3)	7.3 (6.4,8.1)	7.3 (6.6, 8.4)
HbA_{1c} (%)	6.5 (6.1, 7.1)	6.5 (6.1, 7.2)	6.5 (6.1, 7.1)
HbA_{1c} (mmol/mol)	48.0 (43.0, 54.0)	48.0 (43.0, 55.0)	48.0 (43.0, 54.0)
Diabetes Duration (years)	3.0 (0, 8.0)	2.0 (0, 8.8)	3.0 (0, 8.0)
Taking diabetes medication	445 (68.1)	86 (76.1)	359 (66.5)
BMI (kg/m^2)	29.6 ± 4.7	30.5 ± 5.2	29.5 ± 4.5
Waist circumference(cm)	105 ± 12.5	107 ± 13.6	104 ± 12.2
Office systolic blood pressure (mmHg)	140 ± 17.4	139 ± 17.0	140 ± 17.5
Office diastolic blood pressure (mmHg)	77.2 ± 9.7	77.1 ± 10.6	77.2 ± 9.6
Taking antihypertensive medication	441 (67.5)	83 (73.5)	358 (66.3)
Serum cholesterol HDL-cholesterol (mmol/l)	1.3 ± 0.4	1.3 ± 0.4	1.3 ± 0.4
Serum cholesterol LDL-cholesterol (mmol/l)	2.4 ± 0.9	2.4 ± 0.9	2.4 ± 0.9
Serum total cholesterol (mmol/l)	4.5 ± 1.0	4.5 ± 1.0	4.5 ± 1.0
Smoking categories			
Current	90 (13.8)	23 (20.4)	67 (12.4)
Former	357 (54.7)	61 (54.0)	296 (54.8)
Never	206 (31.5)	29 (25.7)	177 (32.8)
Alcohol consumption status[c]			
High	118 (18.1)	23 (20.4)	95 (17.6)
Low	373 (57.1)	59 (52.2)	314 (58.1)
None	162 (24.8)	31 (27.4)	131 (24.3)
EMG composite score			
0, No abnormality	454 (69.5)	390 (72.2)	64 (56.6)
1, Mild abnormality	159 (24.3)	124 (23.0)	35 (31.0)
2, Moderate abnormality	32 (4.9)	20 (3.7)	12 (10.6)
3, Severe abnormality	8 (1.2)	6 (1.1)	2 (1.8)

Demographics and clinical characteristics for all participants with type 2 diabetes, stratified by presence of neuropathic pain. Data are *n* (%), mean ± SD or median (IQR) as appropriate based on variable type and distribution.

Neuropathic pain was defined as a DN4 interview score ≥3

[a]Sex was self-reported by participants at enrolment.

[b]eGFR was calculated using the CKD-EPI equations (with both serum creatinine and cystatin C)

[c]High alcohol consumption was defined as >7 units/week for women and >14 units/week for men

ESM Table 5 EMG and WF-CCM results grouped by neuropathic pain

Variable	Total (*N*=3425)	Neuropathic pain (*N*=306)	No Neuropathic Pain (*N*=3119)
Sural nerve, amplitude (μV)	6.90 (4.10, 11.3)	5.90 (3.10, 9.70)	7.00 (4.20, 11.5)
Tibial nerve, stimulation site ankle, amplitude (mV)	9.81 ± 4.11	8.92 ± 4.20	9.89 ± 4.09
EMG composite score			
0, No abnormality	2749 (80.3)	211 (68.95)	2538 (81.37)
1, Mild abnormality	588 (17.2)	73 (23.86)	515 (16.51)
2, Moderate abnormality	69 (2.0)	17 (5.56)	52 (1.67)
3, Severe abnormality	19 (0.6)	5 (1.63)	14 (0.45)
Corneal nerve fibre length in mm/mm^2	15.0 ± 4.41	14.8 ± 4.53	15.0 ± 4.40
Corneal nerve fibre density in (number of corneal nerve fibres/mm^2)	79.8 ± 24.4	78.6 ± 24.6	79.9 ± 24.3
Corneal nerve branch density in (number of corneal nerve branches/mm^2)	67.3 (44.7, 97.0)	68.1 (45.6, 90.6)	67.2 (44.5, 97.3)
Corneal nerve fractal dimension	1.36 (1.29, 1.41)	1.36 (1.29, 1.41)	1.36 (1.29, 1.41)
Corneal nerve tortuosity (absolute)	3.07 ± 0.54	3.08 ± 0.55	3.07 ± 0.54

Nerve conduction study results and WF-CCM results for all participants in the study, stratified by presence of neuropathic pain. Data are *n* (%), mean ± SD or median (IQR) as appropriate based on variable type and distribution.

Neuropathic pain was defined as a DN4 interview score ≥3

ESM Table 6 EMG Amplitude and WF-CCM results grouped by degree of EMG abnormalities

Variable	Total (*N*=3425)	No EMG abnormalities (*N*=2749)	Mild EMG abnormalities (*N*=588)	Moderate EMG abnormalities (*N*=69)	Severe EMG abnormalities (*N*=19)
Sural nerve, amplitude (μV)	6.90 (4.10, 11.3)	7.80 (5.00, 12.1)	3.60 (0, 7.30)	0 (0, 3.03)	0 (0, 0)
Tibial nerve, stimulation site ankle, amplitude (mV)	9.81 ± 4.11	10.4 ± 3.84	7.64 ± 4.13	3.38 ± 3.09	1.44 ± 1.11
Corneal nerve fibre length in mm/mm^2	15.0 ± 4.41	15.1 ± 4.42	14.8 ± 4.32	14.2 ± 4.62	13.6 ± 4.20
Corneal nerve fibre density in (number of corneal nerve fibres/mm^2)	79.8 ± 24.4	80.0 ± 24.4	79.6 ± 24.2	76.2 ± 25.2	70.6 ± 21.3
Corneal nerve branch density in (number of corneal nerve branches/mm^2)	67.3 (44.7, 97.0)	68.0 (44.8, 97.3)	65.9 (45.3, 95.4)	59.2 (37.5, 94.1)	56.7 (43.2, 76.9)
Corneal nerve fractal dimension	1.36 (1.29, 1.41)	1.36 (1.29, 1.41)	1.35 (1.29, 1.40)	1.33 (1.28, 1.39)	1.27 (1.20, 1.41)
Corneal nerve tortuosity (absolute)	3.07 ± 0.54	3.08 ± 0.54	3.04 ± 0.53	3.09 ± 0.55	3.10 ± 0.47

Nerve conduction study results and WF-CCM results for all participants in the study, stratified by degree of EMG abnormalities. Data are *n* (%), mean ± SD or median (IQR) as appropriate based on variable type and distribution.

Neuropathic pain was defined as a DN4 interview score ≥3

ESM Table 7 Associations of EMG with corneal nerve fibre parameters – Normal glucose metabolism

Model	CNFL (mm/mm^2)	CNFD ($fibres/mm^2$)	CNBD ($branches/mm^2$)	Corneal nerve fractal dimension	Tortuosity
**Mild EMG abnormalities vs no EMG abnormalities**					
Crude model	-0.13 (-0.64, 0.37)	0.64 (-2.13, 3.41)	0.94 (-3.67, 5.54)	-0.01 (-0.02, 0.00)	-0.05 (-0.11, 0.01)
Model 1	-0.10 (-0.60, 0.40)	0.71 (-2.06, 3.48)	1.12 (-3.48, 5.71)	-0.01 (-0.02, 0.00)	-0.05 (-0.11, 0.02)
Model 2	-0.04 (-0.56, 0.48)	1.30 (-1.59, 4.19)	1.82 (-3.00, 6.63)	-0.01 (-0.02, 0.00)	-0.06 (-0.12, 0.01)
**Moderate EMG abnormalities vs no EMG abnormalities**					
Crude model	-0.42 (-2.18, 1.34)	-1.45 (-11.12, 8.23)	-1.89 (-17.98, 14.19)	0.01 (-0.03, 0.05)	0.04 (-0.18, 0.26)
Model 1	-0.43 (-2.18, 1.32)	-1.46 (-11.14, 8.21)	-1.94 (-18.00, 14.12)	0.01 (-0.03, 0.05)	0.04 (-0.18, 0.26)
Model 2	0.03 (-1.79, 1.86)	0.32 (-9.84, 10.48)	0.60 (-16.31, 17.51)	0.01 (-0.03, 0.05)	0.07 (-0.15, 0.30)
**Severe EMG abnormalities vs no EMG abnormalities**					
Crude model	-1.81 (-5.31, 1.68)	-10.16 (-29.42, 9.11)	-16.94 (-48.97, 15.08)	-0.09 (-0.16, -0.02) *	0.07 (-0.36, 0.51)
Model 1	-1.76 (-5.24, 1.72)	-10.05 (-29.30, 9.21)	-16.65 (-48.62, 15.32)	-0.09 (-0.16, -0.02) *	0.09 (-0.35, 0.52)
Model 2	-2.27 (-6.06, 1.53)	-14.00 (-35.11, 7.12)	-25.07 (-60.21, 10.08)	-0.11 (-0.19, -0.04) **	0.06 (-0.41, 0.53)

Values are unstandardised β (95% CI)

Multivariable linear regression was performed. Model 1 was adjusted for WF-CCM lag time; model 2 was adjusted for WF-CCM lag time, age, sex, BMI, height and skin temperature.

Asterisks indicate values that are statistically significant (* = $p<0.05$, ** = $p<0.01$, *** = $p<0.001$).

ESM Table 8 Associations of EMG with corneal nerve fibre parameters – Prediabetes

Model	CNFL (mm/mm^2)	CNFD ($fibres/mm^2$)	CNBD ($branches/mm^2$)	Corneal nerve fractal dimension	Tortuosity
**Mild EMG abnormalities vs no EMG abnormalities**					
Crude model	0.03 (-1.00, 1.06)	1.26 (-4.60, 7.11)	0.15 (-9.19, 9.48)	0.01 (-0.01, 0.04)	-0.01 (-0.13, 0.11)
Model 1	0.20 (-0.81, 1.21)	1.95 (-3.85, 7.75)	1.38 (-7.84, 10.59)	0.02 (-0.00, 0.04)	0.00 (-0.12, 0.12)
Model 2	0.56 (-0.47, 1.59)	2.98 (-3.02, 8.98)	3.28 (-6.27, 12.84)	0.02 (0.00, 0.04) *	0.04 (-0.09, 0.16)
**Moderate EMG abnormalities vs No EMG abnormalities**					
Crude model	0.14 (-2.32, 2.60)	-0.22 (-14.19, 13.75)	-0.47 (-22.74, 21.80)	-0.01 (-0.06, 0.05)	0.13 (-0.16, 0.41)
Model 1	0.26 (-2.15, 2.67)	0.27 (-13.54, 14.08)	0.40 (-21.54, 22.35)	-0.00 (-0.05, 0.05)	0.14 (-0.15, 0.42)
Model 2	0.49 (-2.02, 3.00)	0.58 (-14.05, 15.21)	1.28 (-22.01, 24.57)	-0.00 (-0.06, 0.05)	0.18 (-0.12, 0.48)
**Severe EMG abnormalities vs No EMG abnormalities**					
Crude model	1.01 (-2.92, 4.94)	-4.35 (-26.66, 17.97)	-3.45 (-39.01, 32.12)	0.01 (-0.08, 0.09)	0.31 (-0.15, 0.76)
Model 1	0.52 (-3.34, 4.36)	-6.43 (-28.51, 15.66)	-7.16 (-42.25, 27.93)	-0.00 (-0.08, 0.08)	0.27 (-0.19, 0.72)
Model 2	2.35 (-2.58, 7.28)	0.80 (-27.95, 29.55)	8.30 (-37.49, 54.09)	0.08 (-0.03, 0.18)	0.37 (-0.22, 0.96)

Values are unstandardised β (95% CI)
Multivariable linear regression was performed. Model 1 was adjusted for WF-CCM lag time; model 2 was adjusted for WF-CCM lag time, age, sex, BMI, height and skin temperature.
Asterisks indicate values that are statistically significant (* = p<0.05, ** = p<0.01, *** = p<0.001).

ESM Table 9 Associations of EMG with corneal nerve fibre parameters – Type 2 diabetes

Model	CNFL (mm/mm^2)	CNFD ($fibres/mm^2$)	CNBD ($branches/mm^2$)	Corneal nerve fractal dimension	Tortuosity
**Mild EMG abnormalities vs no EMG abnormalities**					
Crude model	-0.42 (-1.23, 0.39)	-2.20 (-6.65, 2.24)	-3.51 (-10.80, 3.79)	-0.00 (-0.02, 0.02)	0.03 (-0.07, 0.12)
Model 1	-0.30 (-1.09, 0.48)	-1.73 (-6.13, 2.65)	-2.68 (-9.88, 4.52)	-0.00 (-0.02, 0.02)	0.04 (-0.05, 0.14)
Model 2	-0.23 (-1.06, 0.60)	-1.05 (-5.70, 3.59)	-2.16 (-9.79, 5.47)	-0.00 (-0.02, 0.02)	0.03 (-0.07, 0.13)
**Moderate EMG abnormalities vs no EMG abnormalities**					
Crude model	-0.90 (-2.50, 0.71)	-4.74 (-13.56, 4.09)	-7.56 (-22.04, 6.93)	-0.02 (-0.06, 0.02)	-0.01 (-0.20, 0.18)
Model 1	-1.10 (-2.66, 0.46)	-5.53 (-14.24, 3.19)	-8.96 (-23.23, 5.32)	-0.02 (-0.06, 0.01)	-0.03 (-0.22, 0.15)
Model 2	-0.93 (-2.55, 0.69)	-4.10 (-13.17, 4.97)	-7.80 (-22.69, 7.10)	-0.02 (-0.05, 0.02)	-0.04 (-0.23, 0.15)
**Severe EMG abnormalities vs no EMG abnormalities**					
Crude model	-1.95 (-5.07, 1.18)	-9.23 (-26.43, 7.98)	-16.28 (-44.52, 11.97)	-0.03 (-0.10, 0.04)	-0.13 (-0.50, 0.23)
Model 1	-2.19 (-5.24, 0.86)	-10.19 (-27.18, 6.80)	-17.98 (-45.81, 9.85)	-0.03 (-0.10, 0.04)	-0.16 (-0.52, 0.19)
Model 2	-2.07 (-5.61, 1.47)	-6.33 (-26.16, 13.49)	-12.57 (-45.12, 19.99)	-0.05 (-0.13, 0.03)	-0.33 (-0.75, 0.08)

Values are unstandardised β (95% CI)
Multivariable linear regression was performed. Model 1 was adjusted for WF-CCM lag time; model 2 was adjusted for WF-CCM lag time, age, sex, BMI, height and skin temperature.

ESM Table 10 Associations of AXONAL DEGENERATION with corneal nerve fibre parameters

Model	CNFL (mm/mm^2)	CNFD (fibres/mm^2)	CNBD (branches/mm^2)	Corneal nerve fractal dimension	Tortuosity
Crude model	-0.51 (-0.78, -0.24) ***	-1.56 (-3.04, -0.08) *	-3.08 (-5.51, -0.64) *	-0.01 (-0.02, -0.01) ***	-0.05 (-0.08, -0.01) **
Model 1	-0.53 (-0.79, -0.27) ***	-1.63 (-3.10, -0.15) *	-3.21 (-5.63, -0.79) **	-0.02 (-0.02, -0.01) ***	-0.05 (-0.08, -0.02) **
Model 2	-0.40 (-0.68, -0.12) **	-0.95 (-2.51,0.62)	-2.35 (-4.93, 0.23)	-0.01 (-0.01, 0.00) **	-0.05 (-0.08, -0.02) **

Values are unstandardised β (95% CI)

Multivariable linear regression was performed. Model 1 was adjusted for WF-CCM lag time; model 2 was adjusted for WF-CCM lag time, age, sex, BMI, height and skin temperature.

Asterisks indicate values that are statistically significant (* = p<0.05, ** = p<0.01, *** = p<0.001).

ESM Table 11 Associations of sural nerve amplitude with corneal nerve fibre parameters

Model	CNFL (mm/mm^2)	CNFD ($fibres/mm^2$)	CNBD ($branches/mm^2$)	Corneal nerve fractal dimension	Tortuosity
Crude model	0.05 (0.02 0.07) ***	0.12 (-0.02 0.25)	0.17 (-0.06 0.39)	0.001 (0.00 0.001) *	0.01 (0.00 0.01) ***
Model 1	0.05 (0.03 0.08) ***	0.14 (0.00 0.27) *	0.21 (-0.02 0.43)	0.00 (0.00 0.001) **	0.01 (0.00 0.01) ***
Model 2	0.02 (-0.00 0.05)	0.01 (-0.14 0.15)	0.02 (-0.22 0.27)	0.00 (0.00 0.001)	0.01 (0.00 0.01) ***

Values are unstandardised β (95% CI)
Multivariable linear regression was performed. Model 1 was adjusted for WF-CCM lag time; model 2 was adjusted for WF-CCM lag time, age, sex, BMI, height and skin temperature.
Asterisks indicate values that are statistically significant (* = $p<0.05$, ** = $p<0.01$, *** = $p<0.001$).

ESM Table 12 Associations of tibial nerve amplitude with corneal nerve fibre parameters

Model	CNFL (mm/mm^2)	CNFD ($fibres/mm^2$)	CNBD ($branches/mm^2$)	Corneal nerve fractal dimension	Tortuosity
Crude model	0.08 (0.04 0.11) ***	0.30 (0.10 0.51) **	0.52 (0.18 0.86) **	0.002 (0.001 0.002) ***	0.00 (0.00 0.01)
Model 1	0.07 (0.03 0.11) ***	0.28 (0.07 0.48) **	0.47 (0.13 0.81) **	0.001 (0.001 0.002) ***	0.00 (-0.00 0.01)
Model 2	0.01 (-0.03 0.05)	0.04 (-0.20 0.27)	0.16 (-0.22 0.54)	0.00 (-0.001 0.001)	-0.001 (-0.01 0.00)

Values are unstandardised β (95% CI)

Multivariable linear regression was performed. Model 1 was adjusted for WF-CCM lag time; model 2 was adjusted for WF-CCM lag time, age, sex, BMI, height and skin temperature.

Asterisks indicate values that are statistically significant (* = $p<0.05$, ** = $p<0.01$, *** = $p<0.001$).

ESM Table 13 Associations of neuropathic pain with corneal nerve fibre parameters – adjusted for PAIN MEDICATIONS in model 3

Model	CNFL (mm/mm^2)	CNFD (fibres/mm^2)	CNBD (branches/mm^2)	Corneal nerve fractal dimension	Tortuosity
Crude model	-0.19 (-0.71 0.33)	-1.35 (-4.21 1.52)	-0.54 (-5.24 4.17)	1.34 (-0.01 0.01)	0.01 (-0.05 0.07)
Model 1	-0.18 (-0.69 0.33)	-1.32 (-4.17 1.53)	-0.49 (-5.17 4.20)	0.00 (-0.01 0.01)	0.01 (-0.05 0.07)
Model 2	-0.06 (-0.57 0.45)	-1.15 (-4.00 1.71)	-0.22 (-4.92 4.48)	0.00 (-0.01 0.01)	0.02 (-0.04 0.08)
Model 3	0.04 (-0.49 0.57)	-0.94 (-3.91 2.03)	0.66 (-4.23 5.56)	0.00 (-0.01 0.02)	0.03 (-0.03 0.10)

Values are unstandardised β (95% CI)
Multivariable linear regression was performed. Model 1 was adjusted for WF-CCM lag time; model 2 was adjusted for WF-CCM lag time, age, sex, BMI, and height; model 3 was adjusted for WF-CCM lag time, age, sex, BMI, height, EMG, and skin temperature.

ESM Table 14 Associations of EMG with corneal nerve fibre parameters – excluding outliers (n=147)

Model	CNFL (mm/mm^2)	CNFD (fibres/mm^2)	CNBD (branches/mm^2)	Corneal nerve fractal dimension	Tortuosity
**Mild EMG abnormalities vs No EMG abnormalities**					
Crude model	-0.20 (-0.59, 0.18)	0.18 (-1.98, 2.34)	0.06 (-3.54, 3.66)	-0.01 (-0.01, 0.00)	-0.03 (-0.08, 0.02)
Model 1	-0.13 (-0.51, 0.26)	0.41 (-1.74, 2.57)	0.56 (-3.03, 4.15)	0.00 (-0.01, 0.00)	-0.02 (-0.07, 0.03)
Model 2	0.02 (-0.38, 0.42)	1.20 (-1.06, 3.46)	1.62 (-2.15, 5.39)	0.00 (-0.01, 0.01)	-0.03 (-0.07, 0.02)
**Moderate EMG abnormalities vs No EMG abnormalities**					
Crude model	-0.91 (-1.94, 0.11)	-3.98 (-9.72, 1.76)	-6.42 (-15.99, 3.15)	-0.02 (-0.04, 0.00) *	0.00 (-0.13, 0.12)
Model 1	-0.90 (-1.92, 0.12)	-3.94 (-9.66, 1.79)	-6.33 (-15.86, 3.20)	-0.02 (-0.04, 0.00) *	0.00 (-0.13, 0.12)
Model 2	-0.51 (-1.56, 0.53)	-2.25 (-8.18, 3.68)	-4.04 (-13.94, 5.86)	-0.02 (-0.03, 0.00)	0.01 (-0.12, 0.14)
**Severe EMG abnormalities vs No EMG abnormalities**					
Crude model	-0.85 (-2.87, 1.16)	-7.03 (-18.32, 4.26)	-11.06 (-29.89, 7.77)	-0.03 (-0.07, 0.01)	0.09 (-0.16, 0.34)
Model 1	-0.90 (-2.90, 1.10)	-7.18 (-18.43, 4.08)	-11.38 (-30.12, 7.36)	-0.03 (-0.07, 0.00)	0.08 (-0.17, 0.32)
Model 2	-0.41 (-2.78, 1.96)	-3.81 (-17.24, 9.62)	-6.72 (-29.14, 15.70)	-0.03 (-0.07, 0.02)	0.01 (-0.29, 0.30)

Values are unstandardised β (95% CI)
Multivariable linear regression was performed. Model 1 was adjusted for WF-CCM lag time; model 2 was adjusted for WF-CCM lag time, age, sex, BMI, height and skin temperature.
Asterisks indicate values that are statistically significant (* = p<0.05, ** = p<0.01, *** = p<0.001).

ESM Table 15 Associations of neuropathic pain with corneal nerve fibre parameters – excluding outliers (*n*=147)

Model	CNFL (mm/mm^2)	CNFD (fibres/mm^2)	CNBD (branches/mm^2)	Corneal nerve fractal dimension	Tortuosity
Crude model	-0.06 (-0.57, 0.45)	-0.89 (-3.75, 1.96)	0.34 (-4.42, 5.10)	0.00 (-0.01, 0.01)	0.04 (-0.03, 0.10)
Model 1	-0.06 (-0.57, 0.44)	-0.91 (-3.76, 1.93)	0.30 (-4.44, 5.04)	0.00 (-0.01, 0.01)	0.04 (-0.03, 0.10)
Model 2	0.03 (-0.47, 0.54)	-0.83 (-3.68, 2.03)	0.43 (-4.33, 5.19)	0.01 (0.00, 0.02)	0.05 (-0.02, 0.11)
Model 3	0.17 (-0.35, 0.69)	-0.51 (-3.46, 2.44)	1.52 (-3.41, 6.45)	0.01 (0.00, 0.02)	0.07 (0.000, 0.13) *

Values are unstandardised β (95% CI)
Multivariable linear regression was performed. Model 1 was adjusted for WF-CCM lag time; model 2 was adjusted for WF-CCM lag time, age, sex, BMI, and height; model 3 was adjusted for WF-CCM lag time, age, sex, BMI, height, EMG, and skin temperature.
Asterisks indicate values that are statistically significant (* = p<0.05, ** = p<0.01, *** = p<0.001).

Figures

ESM Figure 1

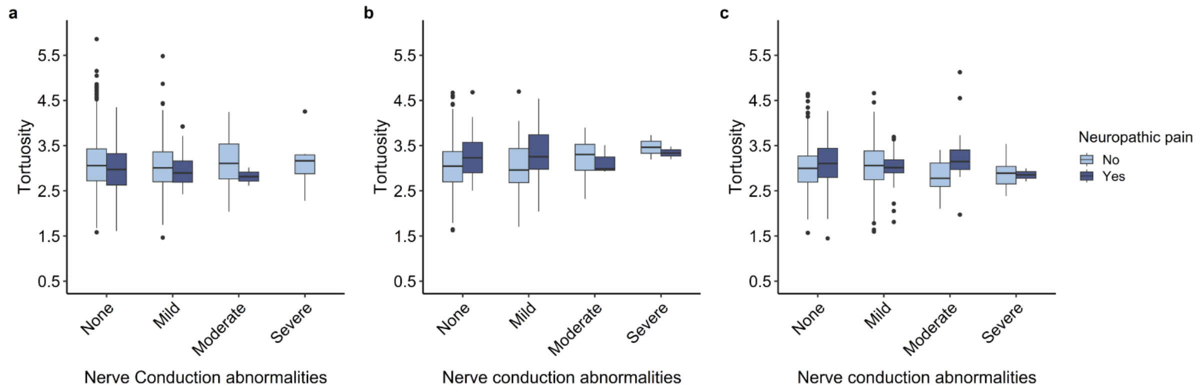

ESM Fig 1 Corneal nerve fibre tortuosity across groups stratified by degree of EMG abnormalities and pain status for participants with normal glucose metabolism (a), prediabetes (b) and type 2 diabetes mellitus (c). Nerve conduction abnormalities on EMG were classified into four severity levels: none, mild, moderate, and severe. No participants with normal glucose metabolism had severe EMG abnormalities in combination with neuropathic pain. Boxplots show median (horizontal line), interquartile range (box), and whiskers extending to 1.5x the interquartile range, dots represent outliers. Pairwise comparison between the group with and without pain were performed using Mann Whitney U-test, significant differences were observed in Fig. 2b (none, p = 0.011) and Fig. 2c (moderate, p = 0.036); all other comparisons were not significant.

ESM Figure 2

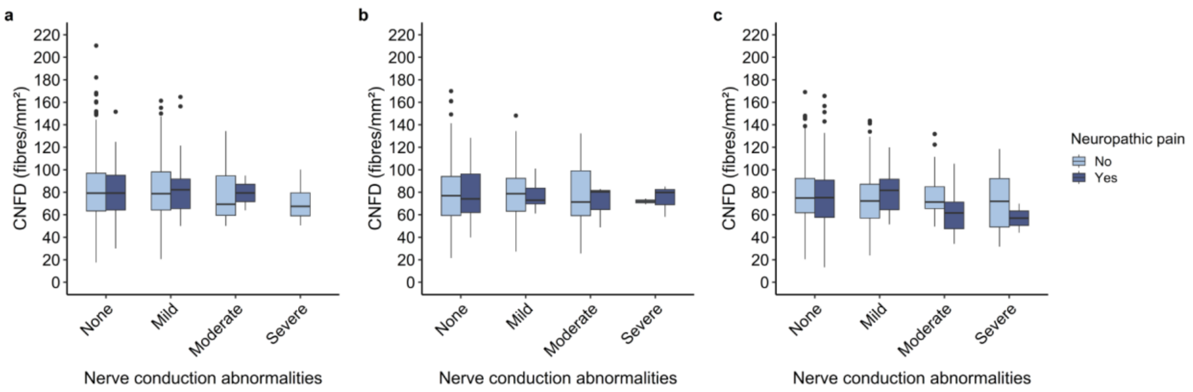

ESM Fig 2 CNFD across groups stratified by degree of EMG abnormalities and pain status for participants with normal glucose metabolism (a), prediabetes (b) and type 2 diabetes mellitus (c). Nerve conduction abnormalities on EMG were classified into four severity levels: none, mild, moderate, and severe. No participants with normal glucose metabolism had severe EMG abnormalities in combination with neuropathic pain. Boxplots show median (horizontal line), interquartile range (box), and whiskers extending to 1.5x the interquartile range, dots represent outliers. Pairwise comparison between the group with and without pain were performed using Mann Whitney U-test, significant differences were observed Fig. 2c (moderate, $p = 0.048$); all other comparisons were not significant.

ESM Figure 3

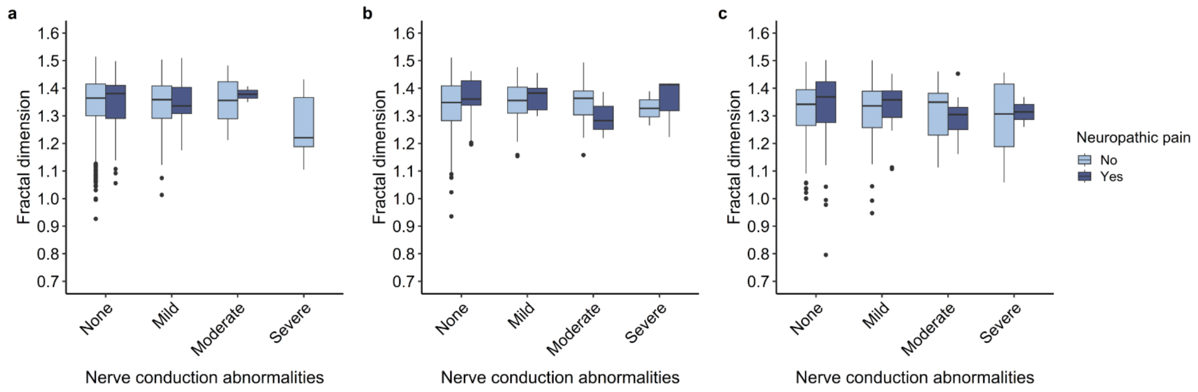

ESM Fig 3 Corneal nerve fibre fractal dimension across groups stratified by degree of EMG abnormalities and pain status for participants with normal glucose metabolism (a), prediabetes (b) and type 2 diabetes mellitus (c). Nerve conduction abnormalities on EMG were classified into four severity levels: none, mild, moderate, and severe. No participants with normal glucose metabolism had severe EMG abnormalities in combination with neuropathic pain. Boxplots show median (horizontal line), interquartile range (box), and whiskers extending to 1.5x the interquartile range, dots represent outliers. Pairwise comparison between the group with and without pain were performed using Mann Whitney U-test, no statistically significant differences were observed.

ESM Figure 4

Interactions between diabetes and exposures

a

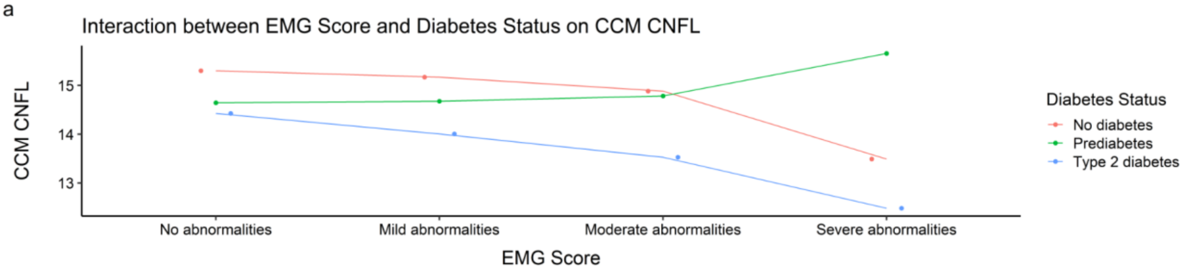

b

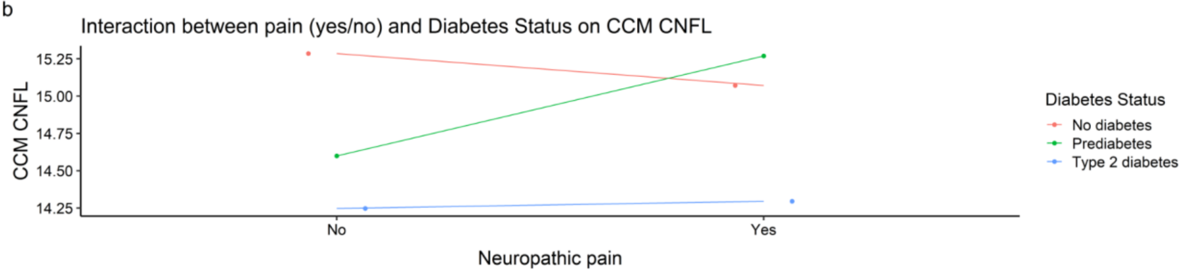

ESM Figure 5

Interactions between sex and exposures

a

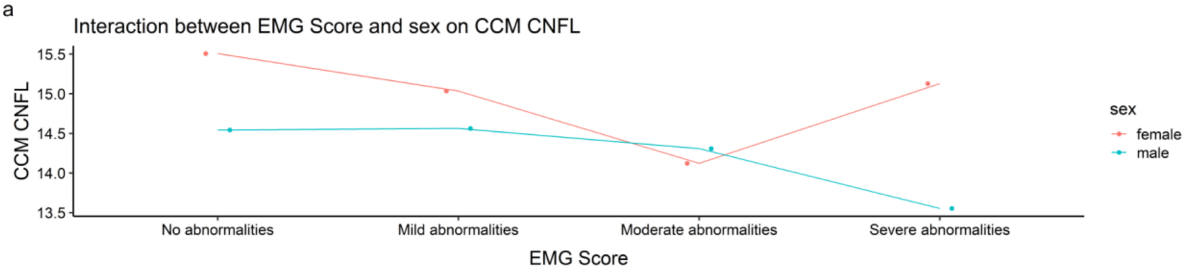

b

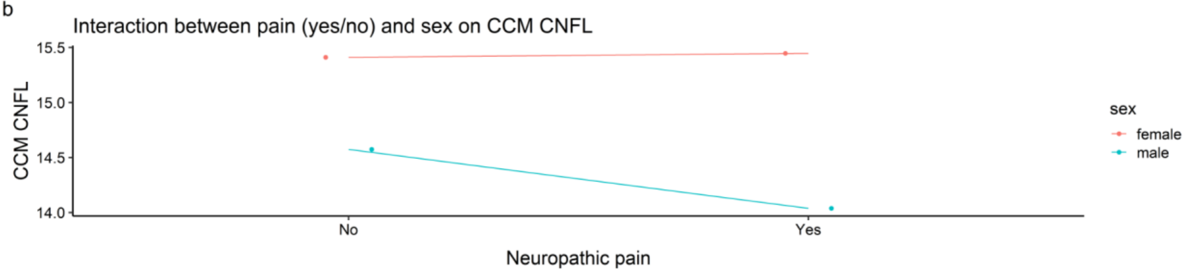

Supplement: Supplementary file 1 — Supplementary file1 (PDF 905 KB) [file 125_2026_6676_MOESM1_ESM.pdf]
